# Supplementary material for: Experimental vulnerability analysis of QKD based on attack ratings
Source: Sci Rep. 2021 May 5;11:9564. doi: 10.1038/s41598-021-87574-4 (PMC8100156; doi:10.1038/s41598-021-87574-4)
Supplement: Supplementary file 1 — Supplementary Information. [file 41598_2021_87574_MOESM1_ESM.pdf]

Experimental vulnerability analysis of QKD based on attack ratings (Supplementary Information)

Rupesh Kumar[1], Francesco Mazzoncini [2], Hao Qin [3], and Romain Alléaume[2]

[1]Quantum Communications Hub, Department of Physics, University of York, Heslington, YO10 5DD, United Kingdom

[2] Télécom Paris-LTCI, Institut Polytechnique de Paris, 19 Place Marguerite Perey, 91120 Palaiseau, France.

[3]CAS Quantum Network Co., Ltd No. 99 Xiupu Rd, Pudong New District, Shanghai 201315, China

In this supplementary material, we consider a relaxed security setting, where channel loss is not precalibrated and actively monitored, by Alice and Bob as it was the case for the security setting considered in the main text. We complement the experimental study of the saturation attacks (coherent[1] and incoherent[2]) in this more relaxed security setting. This allows us to discuss the interplay between security setting and attack ratings.

Moreover, we provide for completeness the formulas that have been used throughout the main text and supplementary material in order to estimate CV-QKD secure key rates, that are computed in the asymptotic limit (infinite number of channel use) and against collective attacks.

Attack success conditions and channel transmittance monitoring

In the main text, we have considered saturation attack under the following success conditions:

(a) Alice and Bob obtain a positive key rate from their estimated parameters T_{sat} and ξ_{sat}

(b) The estimated channel transmittance T_{sat} must be (approximately) equal to the calibrated channel transmittance T.

The second condition implies that Alice and Bob must securely calibrate (independently of the QKD runs and in particular independently of Eve) the channel transmittance. They must then actively monitor the estimated channel transmittance T_{sat}, throughout the QKD runs, to verify that condition (b) applies. We can denote this security setting as *channel transmittance trusted calibration and active monitoring*.

As illustrated in the main text, condition (b) imposes constraints on Eve attack. In particular, we found out that below a certain transmission distances, Eve cannot successfully mount the saturation attack and verify (a) and (b).

Relaxed setting: unmonitored channel transmittance

It is interesting to study the impact of relaxing condition (b) on the possibility to launch the incoherent or coherent saturation attacks. Eve can then mount saturation attacks as long as the excess noise, ξ_{sat} remains below the null key threshold, i.e. enforce condition (a) only, without being limited by condition (b). Figure1 shows the excess noise and key rate in this relaxed security setting, both for coherent and incoherent attack strategy. Since the relaxed suc-

cess condition imposes no contraints on the estimated transmittance, Eve can mount the attacks in all the transmission distances, provided the excess noise can be biased sufficiently by the saturation attacks. This is not the case for the coherent attack, as can be seed on Figure1(a), that hence results in zero key.

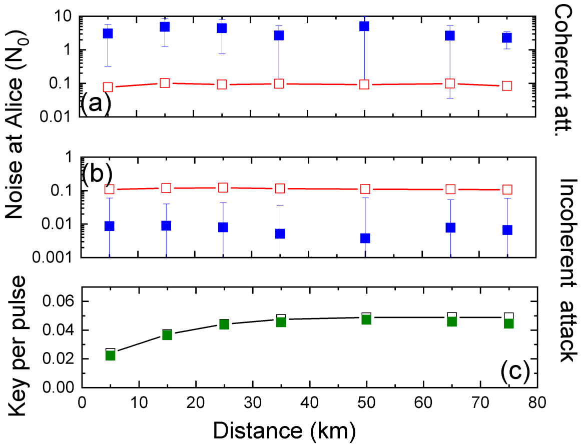

Figure 1: Results.**(a), (b)** excess noise at Alice. Red squares indicate the null key noise threshold and blue squares the estimated values of ξ_{sat}, respectively for coherent and incoherent attack. **(c)** key rate for incoherent attack. Black squares are simulated values of final key per pulse while Green squares are from the experiment. Error bars are one standard deviation of fluctuations among ten smaller data block of size 10^7. Coherent attack returns zero key rate as the excess noise is above the null key threshold.

For the saturation attack based on coherent displacement[1] and incoherent saturation with an external laser,[2] we have estimated, for each transmission distance, the optimal Δ and G such that excess noise falls below the null key threshold, irrespective of the channel transmittance. This is shows in Figure2 (a) for Δ and (b) for G.

Analysis and Attack rating

The *unmonitored channel losses* relaxed security condition clearly results in lower ratings of the possible

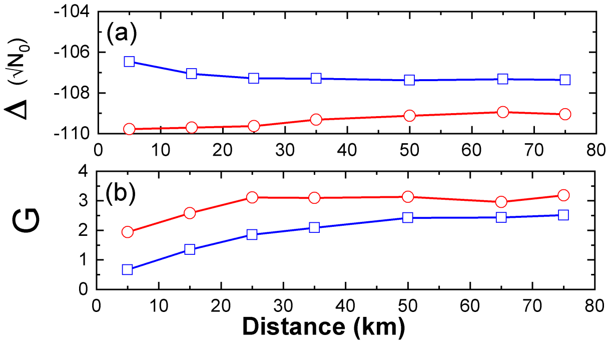

Figure 2: **(a)** Optimal value of Δ and **(b)** Optimal G. at various distance. Red dots for coherent attack and blue squares for incoherent attack. Since Eve does not have to maintain channel transmittance, there exists Δ and G for all transmission distances.

attacks, i.e. attacks easier to implement. One comparison of the impact of the security setting is visible on Figure 3, for coherent and incoherent saturation attacks. The relaxed security setting corresponds to Figure 3 (a). In this security setting, Eve is allowed to operate the attack on a wide range of distances, without restrictions on the value taken by T_{sat}. On the other hand, Figure 3 (b) corresponds to the security setting where channel loss is monitored. The condition $T_{sat} = T$ can only then only be enforced for distances larger than 35 km, limiting the exposition to the attack.

Figure 3 shows the direct comparison of both success conditions. Red solid line shows the channel transmittance with transmission distances, assuming 0.2dB loss per km fibre channel. The simulated (black square)and experimental data (blue solid square) under saturation attack shows: in Figure3 (a) channel transmittance is not maintained as per the relaxed success criteria; in Figure3 (b) channel transmittance can only be maintained after 35km of transmission distance.

If we consider the incoherent saturation attack, its was rated as *Moderate* with an Attack Potential of 14 in the main text. This rating was performed in a security setting where channel loss is actively monitored. As can be seen on Figure 3, we can we can claim

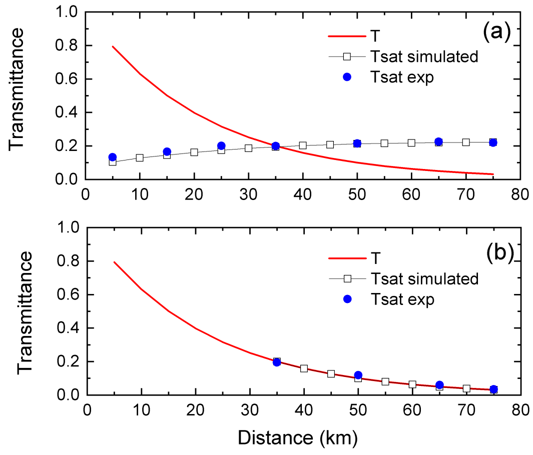

Figure 3: Transmittance at various distance under saturation attack, under two security settings:
(a) T_{sat} is not maintained during the attack where Alice and Bob do not monitor T.
(b) Success condition $T_{sat} = T$ is only maintained for longer transmission distances.

that the tuning of the setup, necessary to successfully perform an attack, will be in general less complex in the relaxed security setting, since the choice of the parameters Δ and G will not be limited by strict constraints any more. This inevitably brings a lower rating, mostly driven by a change in the Window of Opportunity factor (from *Moderate*) to *Easy*). Therefore, the Attack Potential changes to 11, but the qualitative rating remains *Moderate*. This lower complexity is reflected in the absence of a distance limitation for the success of the attack, which allows Eve to operate her attack with less control either of her own hardware, or with respect to Alice and Bob monitoring.

The analysis also illustrates that a QKD protocol and an attack is not only defined by what the players can do during the QKD runs, but are also constrained (and protected) by important additional measures such as calibration and monitoring, that play a direct role in the overall resistance against malicious tampering, and more generally, in the security level that can be provided by the QKD system.

Estimation of secure key rate

In order to evaluate the secure key generation rate, we have considered the security in asymptotic limit, against collective attack. The final secure key rate, in reverse reconciliation, can be written as:

$$K = \beta I_{AB} - \chi_{EB} \tag{1}$$

Here, β is the reconciliation efficiency. I_{AB} is the mutual information between Alice and Bob and χ_{EB} is Eve's the accessible information- Holevo bound,[3] in reverse reconciliation- in which Alice corrects initial quadrature information as per Bob's noisy measurement outcomes. The mutual information between Alice and Bob is:

$$I_{AB} = \frac{1}{2}log_2(1 + \frac{V_A}{1+\chi_{tot}}) \tag{2}$$

Where, χ_{tot} is the total noise, which includes the channel noise $\chi_{line} = (1-T)/T + \xi$ and the detection noise $\chi_{hom} = (1-\eta+v_{ele})/\eta$. Here, η is Bob's detection efficiency and v_{ele} is the electronic noise variance of the homodyne detector.

The Holevo bound for Eve's accessible information can be estimated as:

$$\chi_{EB} = S(\rho_E) - \int p(X_B)S(\rho_{E|B})dX_B \tag{3}$$

where, $S(\rho_E)$ is the Von Neumann entropy of the state that Eve poses, $p(X_B)$ is the probability distribution of Bob's measurements and $\rho_{E|B}$ is Eve's states conditioned on Bob's measurement. The above equation can further simplified into:

$$\chi_{BE} = \sum_{i=1}^{2} G\left(\frac{\lambda_i - 1}{2}\right) - \sum_{i=3}^{5} G\left(\frac{\lambda_i - 1}{2}\right) \tag{4}$$

Where $G(x) = (x+1)\log_2(x+1) - x\log_2 x$, $\lambda_{1,2}$ are the symplectic eigenvalues of the covariance matrix that characterize a joint state ρ_{AB} and $\lambda_{3,4,5}$ are that of the state left after Bob's measurement. One can find the eigenvalues as:

$$\lambda_{1,2}^2 = \frac{1}{2}[A \pm \sqrt{A^2 - 4B}], \tag{5}$$

in which, $A = V^2(1-2T) + 2T + T^2(V+\chi_{line})^2$ and $B = T^2(V\chi_{line}+1)^2$ with $V = V_A+1$. Similarly,

$$\lambda_{3,4}^2 = \frac{1}{2}[C \pm \sqrt{C^2 - 4D}], \quad (6)$$

where, $C = (V\sqrt{B}+T(V+\chi_{line})+A\chi_{hom})/(T(V+\chi_{tot}))$ and $D = \sqrt{B}((V+\sqrt{B}\chi_{hom})/(T(V+\chi_{tot})))$ and the last symplectic eigenvalue λ_5 is 1. Plugging Eq.5 and Eq.6 in Eq.4 we can estimate upper bound of Eve's accessible information and then the final secure key rate from Eq.1.

References

[1] Qin, H., Kumar, R. & Alléaume, R. Quantum hacking: Saturation attack on practical continuous-variable quantum key distribution. *Physical Review A* **94**, 012325 (2016).

[2] Qin, H., Kumar, R., Makarov, V. & Alléaume, R. Homodyne-detector-blinding attack in continuous-variable quantum key distribution. *Phys. Rev. A* **98**, 012312 (2018).

[3] Weedbrook, C. *et al.* Gaussian quantum information. *Reviews of Modern Physics* **84**, 621 (2012).
